# Dollars and Disparities: Unraveling Executive Compensation Patterns in American Non-Profit Healthcare

**DOI:** 10.7759/cureus.70911

**Published:** 2024-10-05

**Authors:** Landon Saipale, Micah Ngatuvai, Jake E Dertinger, Josh Hansen, Mikel Tihista, Richard Purcell

**Affiliations:** 1 Orthopaedic Surgery, Central Michigan University College of Medicine, Saginaw, USA; 2 Orthopaedic Surgery, Nova Southeastern University, Dr. Kiran C. Patel College of Allopathic Medicine, Davie, USA; 3 Orthopaedic Surgery, Texas Tech University Health Sciences Center El Paso, El Paso, USA; 4 Orthopaedic Surgery, William Beaumont Army Medical Center, El Paso, USA

**Keywords:** compensation, compensation practices, executives, hospital finance, hospital revenue, non-profit hospitals, salary

## Abstract

Introduction: This study examines trends in executive compensation at non-profit hospitals across the United States from 2018 to 2022. The research aims to address gaps in the current literature by providing an up-to-date analysis of compensation practices and their relationship to hospital financial metrics.

Methods: A descriptive longitudinal study was conducted using data from Form 990 filings of 20 randomly selected non-profit hospitals across four U.S. regions. Executive compensation data, along with hospital financial metrics, were extracted and analyzed. Statistical analyses included descriptive statistics, non-parametric tests for trend analysis, and correlation studies using Kendall's Tau B.

Results: The median increase in executive compensation over the five-year period was 23.4%, with a median annual increase of 6.2%. Significant positive correlations were found between executive compensation and hospital revenue (τ = 0.676, p < 0.001), net assets (τ = 0.600, p < 0.001), and net income (τ = 0.273, p < 0.001). However, percentage changes in these variables over time were not significantly correlated.

Conclusion: The study reveals significant growth in executive compensation at non-profit hospitals, with notable regional variations. While compensation correlates strongly with hospital financial metrics, the lack of correlation in their respective changes over time suggests a complex relationship. These findings raise important questions about resource allocation and compensation practices in non-profit healthcare institutions, highlighting the need for further research and policy discussions.

## Introduction

Executive compensation in the healthcare sector has become a focal point for both public scrutiny and academic inquiry [1]. In non-profit hospitals, where resources are often perceived as being directed toward patient care and community services, the compensation of top executives can draw significant attention [2]. Understanding how these compensation practices have evolved over time is crucial for maintaining public trust and ensuring financial transparency. As hospitals navigate financial pressures and regulatory changes, executive pay remains a critical area of interest [3,4].

Several studies have examined executive compensation in the healthcare sector, providing a foundation for our analysis. For example, Joynt et al. explored the compensation of nonprofit US hospital Chief Executive Officers (CEOs) using Internal Revenue Service (IRS) Form 990s, finding that larger teaching hospitals and those with advanced technology tended to pay higher salaries [5]. Similarly, Shay and White conducted a systematic review of executive compensation in healthcare, highlighting significant increases over the past decade and noting that financial performance was a minor factor in determining compensation [4]. While these studies provide valuable insights, there is a notable gap in the literature regarding the most recent trends in executive compensation, particularly in the context of rapidly changing healthcare landscapes and economic conditions. Our study aims to address this gap by examining the most recent five-year period across different regions of the United States.

The findings of this study have potential implications for hospital governance, policy-making, and public perception of nonprofit healthcare institutions. By providing a comprehensive and up-to-date analysis of executive compensation practices, this research aims to inform discussions on fair and effective compensation strategies in the nonprofit healthcare sector.

## Materials and methods

Study design and hospital selection

This is a descriptive longitudinal study that assessed five-year (2018-2022) trends in executive compensation for non-profit hospitals. Executives were defined as any individual listed under Schedule VII for the selected form 990, which includes officers, directors, trustees, and the five highest compensated employees. To reduce hospital selection bias and ensure a representative sample of non-profit hospitals across the United States, we utilized stratified random sampling. A list of 100 non-profit hospitals was compiled, after which an Excel random number generator (Microsoft Corporation, Redmond, Washington) was used to select five hospitals from each region, totaling 20 hospitals. We defined four geographical regions according to the U.S. Census Bureau: Northeast, Midwest, South, and West. This sample size was deemed sufficient to provide meaningful insights after a preliminary analysis revealed significant trend increases in executive compensation for 90% of initially examined hospitals.

Inclusion criteria

Only hospitals that had filed Form 990 for the years 2018-2022 were eligible for inclusion in this study. Form 990 is a mandatory tax document for organizations exempt from income tax under section 501(c), providing detailed financial information, including executive compensation.

Data collection and extraction

Executive compensation data were collected using ProPublica’s Nonprofit Explorer, an online resource that provides access to Form 990 filings [6]. Each selected hospital's Form 990 was accessed for the years 2018 through 2022. For each hospital, executive compensation was defined as the sum of Columns D-F in Schedule J, Part II of Form 990, which includes Column D: Reportable compensation from the organization; Column E: Reportable compensation from related organizations; and Column F: Estimated amount of other compensation from the organization and related organizations.

The total compensation for each executive listed in these columns was aggregated to obtain a comprehensive measure of executive compensation for each year. Additional variables such as revenue, net income, and net assets were gathered directly from ProPublica’s website.

Statistical methods

The data collected from 2018 to 2022 were analyzed to identify trends in executive compensation. Descriptive statistics were calculated for each year, along with the percentage change year-over-year. The Shapiro-Wilk test was used to assess the normality of the compensation data distribution. As the data were found to be non-normally distributed, non-parametric tests were employed. The Friedman test was used to assess changes in compensation over the five-year period. The Kruskal-Wallis H test was used to examine differences in compensation between regions. The correlation of total executive compensation to revenue, net income, or net assets was assessed using Kendall’s Tau B correlation. All statistical analyses were performed using Jamovi (Version 2.3; The Jamovi project, 2021, Sydney, Australia), with a significance level set at p < 0.05 [7].

Ethical considerations

This study utilized publicly available data from Form 990 filings and was therefore deemed Institutional Review Board (IRB) exempt.

## Results

Executive compensation overview

We analyzed executive compensation data from Form 990s of 20 non-profit hospitals across four U.S. regions (Northeast, Midwest, South, and West) over a five-year period (2018-2022). Across all hospitals and regions, the median increase in executive compensation over the five-year period was 23.4%. On a year-over-year basis, the median increase in compensation was a 6.2% increase per year (see Table 1).

**Table 1:** Average percent pay increase year-over-year by region

Region	2018-2019	2019-2020	2020-2021	2021-2022
Northeast	2.40%	3.30%	11.20%	6.60%
Midwest	-0.10%	4.60%	10.50%	-2.20%
South	3.50%	12.90%	5.30%	12.00%
West	13.00%	4.90%	1.80%	12.60%
p-value	0.653			

Regional and yearly trends in executive compensation

Analysis of regional trends revealed notable variations in executive compensation growth. Over the five-year period, the Southern region demonstrated the highest median increase at 36.2%, while the Midwest showed the lowest at 18.2%. However, these regional differences in compensation growth were found to be insignificant (p = 0.653). In contrast, our results found a significant increase in salary year-over-year (p = 0.025), suggesting a consistent upward trend in executive compensation across all regions over the study period (see Table 1).

Hospital-specific compensation data

Our analysis identified substantial variations in total executive compensation among individual hospital systems. For each hospital, we calculated the sum of compensation for all listed executives in a given year and then determined the median of these annual totals over the five-year period. The Mayo Clinic in Rochester, Minnesota, emerged as the highest-compensating system, with a median annual total executive compensation of $147,702,999 (range: $139,609,729-$159,102,812) over the five-year period. The Cleveland Clinic in Cleveland, Ohio, followed closely, with a median annual total compensation of $114,534,912 (range: $91,471,656-$126,198,059) (see Table 2).

**Table 2:** Average dollar amount paid to executives year-over-year from 2018 to 2022 Amount in U.S. dollars.

Hospital	Region	2018	2019	2020	2021	2022
Mount Sinai Hospital	Northeast	31,388,620	33,632,488	32,986,060	35,661,749	38,167,862
NewYork-Presbyterian Hospital	Northeast	59,418,803	54,062,263	59,270,890	66,681,875	70,674,066
Yale New Haven Hospital	Northeast	30,581,021	31,971,935	31,782,913	35,338,886	36,340,867
New England Hospital	Northeast	6,100,633	13,047,153	13,861,722	14,171,649	17,981,151
Penn State Health Holy Medical Center	Northeast	11,297,359	9,414,580	8,905,732	11,399,941	10,882,995
Mayo Clinic	Midwest	147,702,999	139,609,729	142,096,639	153,753,812	159,102,812
Cleveland Clinic	Midwest	91,471,656	101,256,127	115,121,058	126,198,059	114,534,912
Indiana University Health	Midwest	29,260,964	25,465,704	23,458,690	27,935,839	22,666,274
University of Wisconsin Medical Foundation	Midwest	16,719,212	17,158,333	20,170,466	20,722,971	24,861,366
The Good Samaritan Hospital	Midwest	16,337,899	17,697,506	14,217,068	19,603,258	19,305,839
Baylor University Medical Center	South	14,018,911	12,839,280	11,571,468	9,170,523	8,902,818
Methodist Hospital	South	34,946,888	37,282,409	40,154,668	41,998,669	45,126,993
St. Josephs Hospital	South	12,882,796	14,064,747	15,782,175	16,184,742	17,548,133
Baptist Health South Florida	South	15,809,385	17,014,700	21,675,700	23,301,542	26,589,622
Vanderbilt University Medical Center	South	21,683,422	21,581,687	26,857,505	31,567,092	38,683,525
Banner - University Medical Center Phoenix	West	37,832,503	44,167,194	40,595,619	45,537,878	48,988,411
Phoenix Children’s Hospital	West	16,957,519	19,670,993	19,272,221	20,297,351	22,442,721
City of Hope	West	13,830,650	19,642,063	22,532,959	21,806,163	30,775,629
Stanford Health Care	West	27,347,636	25,410,004	30,348,526	30,065,029	33,088,717
Cedars-Sinai Medical Center	West	28,630,630	31,924,842	34,958,374	32,612,489	34,029,776

In contrast, some hospital systems reported significantly lower total executive compensation levels. The Penn State Health Holy Medical Center in Hershey, Pennsylvania, had the lowest median annual total executive compensation at $10,882,995 (range: $8,905,732-$11,399,941). Baylor University Medical Center in Dallas, Texas, also reported relatively low compensation, with a median annual total of $11,571,468 (range: $8,902,818-$14,018,911).

Associations between executive compensation and hospital financial metrics

Our analysis revealed significant correlations between executive compensation and key financial metrics of the hospitals studied. We found a strong positive association between executive compensation and hospital revenue (Kendall's Tau B = 0.676, p < 0.001), indicating that hospitals with higher revenues tend to offer higher executive compensation. Similarly, executive compensation showed a strong positive correlation with net assets (Kendall's Tau B = 0.600, p < 0.001) and a moderate positive correlation with net income (Kendall's Tau B = 0.273, p < 0.001).

Interestingly, when we examined the relationships between percent changes in these variables over time, we found no significant correlations. The percentage change in executive compensation was not significantly associated with changes in revenue (Kendall's Tau B = 0.062, p = 0.413), net income (Kendall's Tau B = -0.046, p = 0.547), or net assets (Kendall's Tau B = 0.007, p = 0.927).

## Discussion

Our study reveals significant growth in executive compensation at non-profit hospitals over the past five years, with an overall median increase of 23.4%. This trend is particularly pronounced in certain regions and hospital systems, such as the Southern region, where executives experienced a median five-year salary increase of 36.2%. These findings raise important questions about the factors driving executive compensation in non-profit healthcare institutions.

The strong positive correlations we found between executive compensation and hospital revenue, net assets, and net income suggest that financial performance plays a role in determining executive pay. However, the lack of significant correlations between changes in these financial metrics and changes in executive compensation over time indicates a more complex relationship. This complexity aligns with previous research by Joynt et al. [5] and Shay and White [4], which found little correlation between CEO compensation and hospital performance measures.

Our findings should be considered in the context of broader healthcare spending trends. The significant increase in executive compensation we observed parallels the overall growth in healthcare administrative expenses noted in previous studies [8,9]. For instance, the 6.2% increase in administrative expenses during the COVID-19 pandemic, compared to a mere 0.6% increase in healthcare services, suggests a growing disparity between administrative costs and direct patient care expenses [10].

Of concern, this upward trend in executive compensation stands in stark contrast to the downward pressure on physician reimbursement [11,12]. While our study shows executive pay rising consistently, numerous studies have documented declining physician reimbursement [13-15]. A particularly striking example comes from the field of orthopedic surgery, where inflation-adjusted Medicare reimbursement rates have steadily declined over time [16,17]. Specifically, from 2019 to 2022, reimbursement rates decreased by an average of 24.5% for shoulder and elbow surgeries, 37.1% for hip arthroplasty, and 40.6% for total knee arthroplasty [16,17]. This widening gap between rising executive compensation and falling physician reimbursement raises serious concerns about resource allocation within the healthcare system. Such disparities may have far-reaching implications for healthcare delivery, workforce retention, and ultimately, patient care, particularly in specialties experiencing the most significant declines in reimbursement rates.

This study has several limitations. First, our analysis was based on data from only 20 randomly selected hospitals. While this sample provides valuable insights, a larger sample size would offer more robust and generalizable findings. Future studies should utilize larger sample sizes to confirm these findings and re-assess for regional differences. Second, our reliance on Form 990 filings introduces a data source limitation, as it excludes IRC section 115 organizations, which are not required to file these forms [18]. This limitation may have resulted in the omission of many university-affiliated hospitals from our analysis, potentially skewing our results. Third, the complex tax structures of many healthcare organizations pose a challenge to our analysis. While most organizations file a single Form 990, a subset of institutions, typically those with complex organizational structures, submit multiple forms for different entities or branches. In these cases, our analysis may underestimate total executive compensation, as we cannot reliably aggregate data across separate filings for the same executive. Fourth, our study may be affected by data collection bias, as we relied solely on information publicly available in Form 990 filings. This limited our ability to account for important factors that could influence executive compensation such as market conditions or hospital-specific performance metrics. These characteristics could provide additional insight into the determinants of executive pay in non-profit hospitals. Lastly, it should be noted that the COVID-19 pandemic occurred during the included years and, therefore, could have impacted the results.

Despite these limitations, we believe this is an important study that highlights some of the key financial trends in healthcare. Future studies should address the limitations of this research by expanding the sample size, incorporating a wider range of hospitals, and considering additional factors that may influence executive compensation. Such research will be crucial in informing policy decisions and ensuring that compensation practices in non-profit hospitals align with their mission of providing high-quality, cost-effective healthcare to their communities.

## Conclusions

This study provides valuable insights into the trends of executive compensation in non-profit hospitals across the United States. Our findings reveal significant growth in executive pay over the past five years, with notable regional variations. Furthermore, this growth in executive compensation per year was not significantly correlated to the hospital performance metrics assessed, such as changes in revenue or net assets.

These trends raise important questions about resource allocation, transparency, and accountability in non-profit hospitals. As healthcare costs continue to rise and disparities in compensation between executives and other healthcare professionals widen, there is a pressing need for further research and policy discussions on this topic.
